# The paid sick leave and sickness benefits for universal health coverage: a scoping review

**DOI:** 10.1093/eurpub/ckac131.121

**Published:** 2022-10-25

**Authors:** H Lee, Y Kwon, K Ryu, M Sohn, H Chung

**Affiliations:** 1 Department of Public Health Sciences, Graduate School, Korea University, Seoul, South Korea; 2 BK21FOUR Learning Health Systems, Korea University, Seoul, South Korea; 3 Division of Health and Medical Sciences, Cyber University of Korea, Seoul, South Korea; 4 School of Health Policy and Management, Korea University, Seoul, South Korea

## Abstract

**Background:**

The countries with paid sick leave (PSL) and sickness benefits (SB) mostly provide the benefit coverage to specific categories of workers, which results in health inequalities among employees in COVID-19. The PSL and SB are key factors to achieve universal health coverage (UHC) in that they protect access to healthcare and improve population health. This study attempted to investigate whether the policies helped achieve the UHC when they were expanded.

**Methods:**

This review followed the scoping review protocol of PRISMA-ScR. On April 6, 2021, we extracted the literature using the keywords ‘paid sick leave', ‘sickness benefits', ‘paid sick day', and ‘earned sick leave’ from PubMed and Web of Science and added two studies through hand-search. All articles were written in English. We did not limit the publication date.

**Results:**

Forty-four selected studies were based in four single countries and the European Union. Most of the studies were published after 2010 (84.1%) and were conducted as cross-sectional (72.7%) studies. Not only workers who use PSL and SB but also children whose parents use PSL and SB increased their use of healthcare services and getting flu shots. Also, using PSL and SB decreased their unmet healthcare needs and emergency use. The various health status factors, such as infectious disease incidence, mortality, and presenteeism, also decreased.

**Conclusions:**

The provisions of PSL and SB offer individual and public health benefits by allowing employees and their families to use healthcare services. Group of employees, we can expect similar public health impacts on newly covered groups, thus contributing to achieving the UHC. Since more than 90% of articles are published from the United States, future studies need to evaluate the outcomes of health effects in various European or Asian countries.

**Key messages:**

• The provision of PSL and SB positively affects employees and their families by allowing them to use healthcare services.

• The expansion of PSL and SB contributes to the UHC by guaranteeing indirect medical costs that enable universal access to essential healthcare services.

